# Letter to the Editor: Beyond Rural Versus Urban: The Role of Referral Pathways in Interpreting Outcomes After Left Atrial Appendage Occlusion

**DOI:** 10.1002/clc.70426

**Published:** 2026-09-16

**Authors:** Muhammad Umer Hameed, Abdullah Khan, Awais Mehmood

**Affiliations:** ^1^ Abbottabad International Medical College Abbottabad Pakistan; ^2^ Khyber Medical University Peshawar Pakistan; ^3^ Azra Naheed Medical College Lahore Pakistan; ^4^ Superior University Lahore Pakistan; ^5^ Continental Medical College Lahore Pakistan; ^6^ University of Health Sciences Lahore Pakistan

## Author Contributions

The authors have read and approved the final version of this letter.

## Funding

The authors have nothing to report.

## Ethics Statement

The authors have nothing to report.

## Consent

The authors have nothing to report.

## Conflicts of Interest

The authors declare no conflicts of interest.

To the Editor,

I read with great interest the recent study evaluating inpatient outcomes after left atrial appendage occlusion (LAAO) in rural versus urban hospitals across the United States [1]. The authors report comparable adjusted mortality and complication rates despite marked differences in procedural volume between settings, an important and reassuring finding. I would like to raise several considerations that may help contextualize this comparison.

First, referral pathways may substantially confound the rural–urban comparison. Patients undergoing LAAO at rural hospitals may represent a preselected, lower‐risk population with relatively straightforward left atrial appendage anatomy, whereas patients with more complex morphology, greater comorbidity burden, or higher anticipated procedural risk are more likely to be referred to tertiary urban centers with established structural heart programs [2]. If such informal triage occurred upstream of the procedure, hospital location in this analysis may partly reflect differences in patient selection rather than genuine differences in procedural quality. This mechanism could also explain a notable pattern in the original data: unadjusted complication rates were numerically higher at urban hospitals despite their considerably greater procedural volume. Rather than being paradoxical, this may simply reflect a higher‐risk case mix being channeled toward urban centers, which would elevate raw complication rates independent of operator skill or institutional quality. Consistent with this volume‐related concern, an analysis of the NCDR LAAO Registry found that lower hospital and physician procedural volumes were independently associated with a reduced likelihood of procedural success, underscoring that volume and outcome are not always straightforwardly related to setting alone [3].

Second, the higher frequency of discharge to acute care facilities among rural patients merits further discussion. Although inpatient mortality and procedural complications were comparable after adjustment [1], the greater need for post‐discharge transfer may reflect differences in regional care pathways rather than differences in procedural safety. Rural hospitals may appropriately perform LAAO while relying on affiliated facilities for downstream monitoring, rehabilitation, or management of complex comorbidities [4]. Discharge disposition may therefore reflect regional organization of healthcare delivery and resource allocation rather than adverse procedural outcomes, and this distinction is relevant when evaluating equity in structural heart disease care.

Third, a binary rural‐versus‐urban framework may not fully capture institutional expertise in contemporary LAAO practice. LAAO is increasingly delivered within comprehensive structural heart disease programs alongside transcatheter aortic valve replacement, transcatheter mitral interventions, and other advanced catheter‐based therapies. The availability of a multidisciplinary heart‐valve team, dedicated cardiac imaging specialists, standardized patient‐selection protocols, and experienced peri‐procedural support may influence outcomes more than geographic location per se [5]. Future studies incorporating measures of structural heart program maturity, rather than hospital location alone, may better identify the modifiable institutional factors that drive optimal outcomes.

Administrative databases of the kind used in this study typically lack granular anatomical and referral‐related variables, which limits the ability to fully disentangle case complexity from the hospital setting. Future work would benefit from incorporating referral‐network data, including interhospital transfer patterns and the clinical characteristics of patients referred from rural to urban institutions specifically for LAAO. Where feasible, linking regional referral registries to procedural databases would allow investigators to formally adjust for this selection effect rather than assume it to be negligible.

In summary, the reassuring finding of comparable adjusted outcomes between rural and urban hospitals should be interpreted with appropriate caution, as it may partly reflect effective triage of higher‐risk patients toward urban centers rather than true equivalence in procedural safety across settings. Future investigations accounting for referral pathways and structural heart program infrastructure will be important to clarify this relationship.

## Data Availability

Data sharing is not applicable to this article as no data sets were generated or analyzed during the current study.
